# Disitamab vedotin combined with PD-1 inhibitors for postoperative adjuvant therapy in upper tract urothelial carcinoma: a retrospective exploratory case series

**DOI:** 10.3389/fphar.2026.1952518

**Published:** 2026-09-14

**Authors:** Yongbao Wei, Huaishan Hong, Dewen Zhong, Ruochen Zhang, Zhixuan Guo, Huang Lin, Jun Lin, Tao Li, Jinfeng Wu, Jianhui Chen, Yi Zhang

**Affiliations:** 1 Department of Urology, Shengli Clinical Medical College of Fujian Medical University, Fuzhou University Affiliated Provincial Hospital, Fuzhou, China; 2 Department of Urology, Longyan First Affiliated Hospital of Fujian Medical University, Longyan, Fujian, China; 3 Department of Urology, Fujian Medical University Union Hospital, Fuzhou, China; 4 Department of Urology, The Affiliated People’s Hospital of Fujian University of Traditional Chinese Medicine, Fuzhou, Fujian, China

**Keywords:** antibody-drug conjugate, disitamab vedotin, PD-1 inhibitor, postoperative treatment, upper tract urothelial carcinoma

## Abstract

Upper tract urothelial carcinoma (UTUC) carries a high risk of postoperative recurrence; however, the decline in renal function following radical nephroureterectomy often limits the use of cisplatin-based chemotherapy. While the combination of disitamab vedotin and PD-1 inhibitors has demonstrated antitumor activity in advanced urothelial carcinoma, evidence regarding its use as postoperative adjuvant therapy remains limited. This study retrospectively identified patients with UTUC who underwent radical resection with R0 margins and had no distant metastasis prior to treatment, and who subsequently received disitamab vedotin combined with a PD-1 inhibitor due to unsuitability for or refusal of platinum-based chemotherapy. A total of seven patients were included; all were male, with a median age of 65 years. Pathological analysis confirmed pure urothelial carcinoma without histological variants in all cases. Four patients exhibited high-risk features (≥pT3 stage or lymph node positivity); all cases showed HER2 expression of IHC 2+ or 3+ and PD-L1 expression <1%. All patients completed four cycles of the combination therapy. With a median follow-up of 22.3 months, three recurrences and two deaths occurred, including three cases of local recurrence. The 12-month postoperative recurrence-free survival (RFS) rate was 71.4%, and the median RFS had not yet been reached. Treatment-related adverse events (TRAEs) of any grade and Grade ≥3 TRAEs occurred in four and two patients, respectively; no Grade 4–5 adverse events or treatment-related deaths were observed. These results provided a preliminary description of the practical feasibility and observed safety of this regimen in a specific cohort of postoperative UTUC patients; however, given the very small sample size and the lack of a control group, no conclusions regarding therapeutic efficacy could be drawn, and further prospective studies are required for evaluation.

## 1 Introduction

Upper tract urothelial carcinoma (UTUC) accounts for approximately 5%–10% of all urothelial carcinomas and is characterized by advanced staging at diagnosis and a high risk of postoperative recurrence. Radical nephroureterectomy (RNU) remains the primary treatment for high-risk, non-metastatic UTUC. While the POUT randomized trial confirmed the value of postoperative platinum-based chemotherapy for disease control, the decline in renal function following RNU often renders some patients ineligible for cisplatin therapy (Masson-Lecomte et al., 2025; Birtle et al., 2020). Research findings regarding adjuvant immunotherapy in urothelial carcinoma have been inconsistent, and high-quality evidence specifically for UTUC remains limited (Bellmunt et al., 2021). Consequently, there remains an unmet clinical need for postoperative UTUC patients who are ineligible for or decline platinum-based chemotherapy.

HER2 expression in urothelial carcinoma exhibits significant heterogeneity, with detection rates and clinical significance influenced by factors such as testing methods, scoring criteria, and tumor location (Soria et al., 2017; Scherrer et al., 2022). Disitamab vedotin (DV) is an antibody-drug conjugate (ADC) comprising a humanized anti-HER2 antibody linked to monomethyl auristatin E (MMAE); it has demonstrated antitumor activity in HER2-expressing locally advanced or metastatic urothelial carcinoma (Sheng et al., 2024). MMAE-based ADCs may synergize with immunotherapy through mechanisms such as tumor cell death, antigen release, and enhanced antigen presentation (Iwata et al., 2018), and the combination of DV with PD-1 inhibitors has shown clinical activity in studies of advanced urothelial carcinoma (Zhou et al., 2025; Sheng et al., 2025).

In recent years, evidence regarding perioperative systemic therapy for UTUC has been accumulating, including systematic reviews of postoperative chemotherapy and immunotherapy (Liu et al., 2025), long-term follow-up results of cisplatin-based neoadjuvant chemotherapy (Jayalath et al., 2026), studies on neoadjuvant immunotherapy for cisplatin-ineligible patients (Huang et al., 2025), and explorations of DV combined with immunotherapy in kidney-sparing treatment, metastatic UTUC, and the postoperative setting (Ye et al., 2025; Ng et al., 2025; Wang et al., 2026). However, detailed patient-level data remain limited for patients who have undergone R0 resection, present with no clear distant metastasis prior to postoperative systemic therapy, and receive DV combined with a PD-1 inhibitor due to ineligibility for or refusal of platinum-based chemotherapy. Therefore, this study aims to characterize the actual treatment exposure, patterns of recurrence, individual clinical courses, and safety profile in this specific patient population, rather than to evaluate the comparative efficacy or adverse effects of the regimen versus standard treatment.

## 2 Materials and methods

### 2.1 Study design and patients

This study was a retrospective, exploratory case series utilizing data from a real-world cohort study of postoperative DV combined with PD-1 inhibitors, established across multiple tertiary hospitals in Fujian Province between January 2021 and December 2024. The study was approved by the local ethics committee (K2026-08-029). Case selection was based on predefined variables regarding primary tumor site, complete surgical resection, exposure to postoperative therapy, and availability of follow-up data.

Inclusion criteria were as follows: (1) primary tumor located in the renal pelvis or ureter, confirmed by postoperative pathology as pure urothelial carcinoma without histological variants; (2) completion of curative-intent complete surgical resection with negative margins (R0); (3) absence of definite distant metastasis prior to postoperative systemic therapy; (4) unsuitability for or refusal of platinum-based chemotherapy due to factors such as renal function, comorbidities, tolerability, or patient preference, followed by treatment with DV combined with a PD-1 inhibitor; and (5) availability of data regarding recurrence, death, and follow-up. The sample size was determined by the total number of eligible cases in the database; no formal sample size or power calculations were performed. The detailed patient selection process was shown in Figure 1.

**FIGURE 1:**
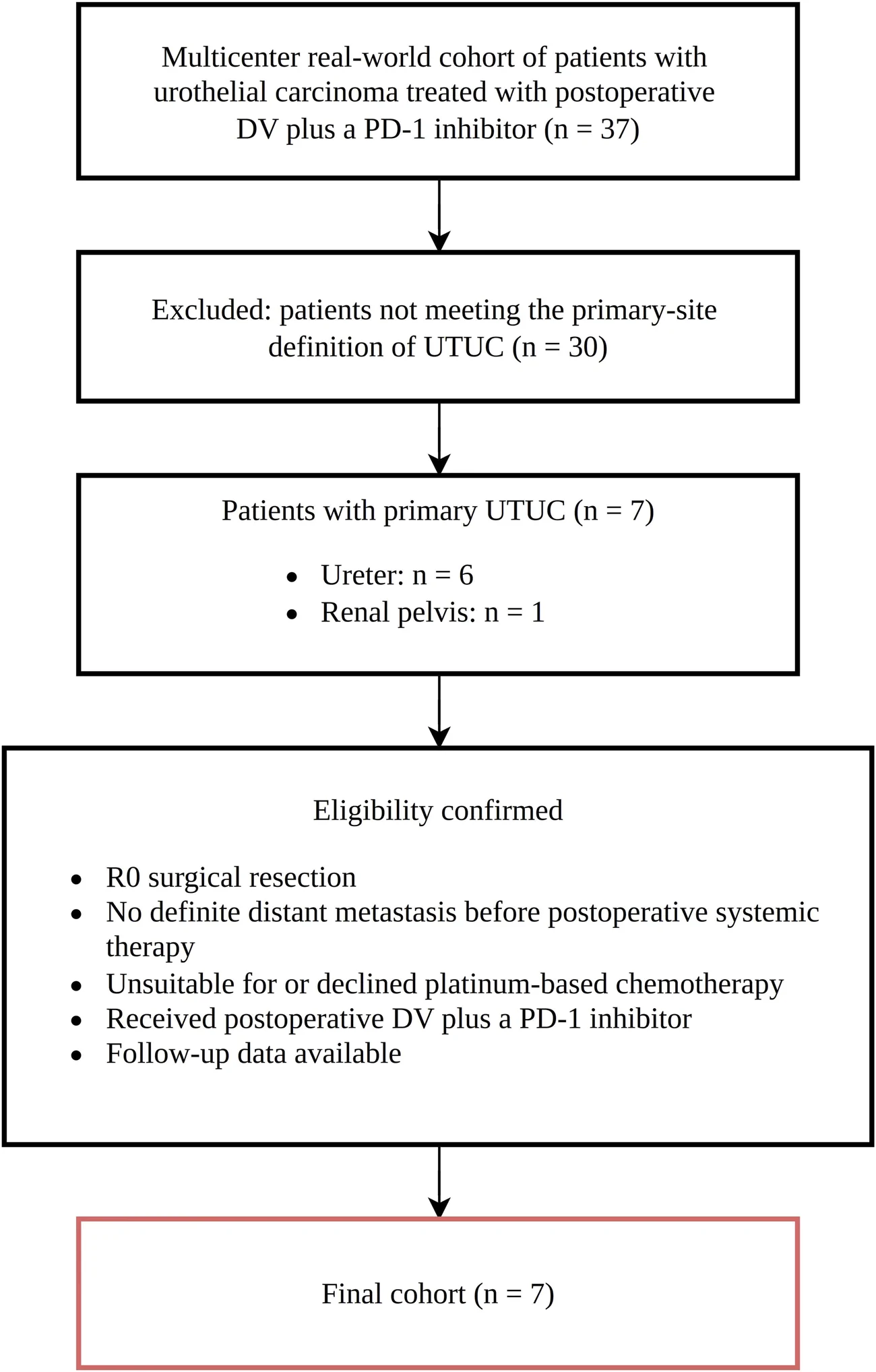
Flow diagram of patient selection from the original multicenter real-world cohort. DV, disitamab vedotin; PD-1, programmed cell death protein 1; UTUC, upper tract urothelial carcinoma; R0, microscopically margin-negative resection.

### 2.2 Pathology and biomarker documentation

HER2 expression was assessed using immunohistochemistry (IHC) and categorized as 0, 1+, 2+, or 3+. The 0–3+ semi-quantitative classification framework used by the participating centers in this study was based on the 2013 American Society of Clinical Oncology/College of American Pathologists (ASCO/CAP) guidelines for HER2 testing in breast cancer (Wolff et al., 2013) and aligned with the HER2 IHC 0–3+ classification system for urothelial carcinoma outlined in the 2021 Chinese expert consensus on HER2 testing in urothelial carcinoma (Tumor Pathology Committee of Chinese Anti-Cancer Association, Expert Committee on Urothelial Carcinoma of Chinese Society of Clinical Oncology, 2021). HER2 results were obtained from routine postoperative pathology reports at each center; no central pathology review or routine fluorescence *in situ* hybridization (FISH) confirmation was performed. Therefore, the IHC 2+/3+ status in this study primarily reflects real-world HER2 expression and the context of treatment selection. PD-L1 expression was evaluated by immunohistochemistry using the Roche VENTANA PD-L1 (SP263) assay system in all seven patients. PD-L1 results were also obtained from routine pathology reports at each center and categorized as <1% or ≥1% based on a 1% threshold.

### 2.3 Treatment, follow-up, and adverse events

The postoperative treatment regimen consisted of intravenous DV combined with a PD-1 inhibitor, administered in 3-week cycles. The initial regimen allowed for the combination of DV (120 mg) with either toripalimab (3 mg/kg) or tislelizumab (200 mg); following the combination phase, PD-1 inhibitor monotherapy maintenance could be continued based on clinical judgment. This study did not pre-specify a uniform PD-1 inhibitor; specific drug selection was based on real-world clinical practice. As this was a retrospective case series, PD-1 inhibitor selection was not randomized, and the original database did not record the rationale for specific drug choices in a standardized or structured manner; consequently, the specific impact of patient clinical status, drug availability, or other clinical factors on drug selection could not be reliably determined. Given the small sample size, this study did not compare the efficacy or safety of toripalimab versus tislelizumab. Similarly, the study protocol did not pre-specify a uniform maintenance therapy strategy following the completion of combination therapy. Decisions regarding whether to continue PD-1 inhibitor monotherapy and the duration of maintenance therapy were individualized based on real-world clinical practice. Because the retrospective database did not systematically record the specific reasons for initiating, omitting, or discontinuing maintenance therapy as standardized structured variables, it was not possible to reliably reconstruct the rationale behind these decisions. Therefore, this study provides only a descriptive report of maintenance therapy exposure rather than analyzing it as a variable of treatment effect. Follow-up was conducted via medical records, outpatient visits, and telephone records; imaging assessments primarily consisted of computed tomography or magnetic resonance imaging, supplemented by bone scans when necessary.

TRAEs were recorded based on the judgment of the treating physician as documented in the medical records and were graded according to the National Cancer Institute Common Terminology Criteria for Adverse Events version 4.0 (Chen et al., 2012). The analysis utilized the highest grade recorded for each patient.

### 2.4 Definition of outcomes

The primary outcome of this study was postoperative recurrence-free survival (RFS), defined as the time from surgery to the first documented recurrence of urothelial carcinoma or death from any cause, whichever occurred first; where source records permitted, the initial recurrence was further characterized as local or distant. Secondary outcomes included overall survival (OS), treatment exposure, TRAEs, and individual clinical courses. OS was defined as the time from surgery to death from any cause; patients who did not experience the event were censored at the date of the last confirmed recurrence-free or survival status. Due to the scarcity of death events, OS data were used only to describe the number of deaths, individual survival status, and follow-up duration; OS rates at fixed time points or median OS were not reported. Surgery was selected as time zero because the time variables in the source database were calculated based on the index surgery date; these outcomes reflect the overall postoperative clinical course.

### 2.5 Statistical analysis

Given the very small sample size and the absence of a control group, statistical analysis was limited to descriptive and exploratory approaches. Continuous variables are presented as medians (ranges), and categorical variables as counts (percentages). For the proportions of recurrence and death, two-sided Clopper-Pearson exact 95% confidence intervals (CIs) were calculated to avoid the instability associated with normal approximations in small samples. Adverse events were summarized descriptively by the number of patients and percentage, based on the highest grade recorded for each patient. As a single patient could experience more than one TRAE, the percentages for different adverse event categories were not mutually exclusive. No imputation was performed for missing data; the denominator for each metric was the number of evaluable patients. Postoperative RFS was estimated using the Kaplan–Meier method (Schemper and Smith, 1996), with 95% CIs for point estimates calculated using Greenwood’s variance formula and log–log transformation. Statistical analysis was performed using Python 3.13.5; Kaplan–Meier estimates utilized statsmodels version 0.14.6, exact binomial confidence intervals were calculated using SciPy 1.17.0, and individual clinical course plots were generated using Matplotlib 3.10.8.

## 3 Results

### 3.1 Patient characteristics

The initial multicenter database included a total of 37 patients with urothelial carcinoma who received postoperative DV combined with a PD-1 inhibitor. Based on predefined criteria regarding the primary tumor site, 30 patients with non-UTUC were excluded, resulting in the final inclusion of 7 patients with UTUC (Figure 1). All included patients were male, with a median age of 65 years (range: 57–77 years) and an Eastern Cooperative Oncology Group performance status score of 0. Primary tumor sites were the ureter (n = 6) and renal pelvis (n = 1) (Table 1). All patients underwent complete curative surgical resection achieving R0 margins; pathological findings showed pure urothelial carcinoma in all cases, with no histological variants observed. Pathological stages included pT2 (n = 4), pT3 (n = 2), and pT4 (n = 1); two patients were lymph node-positive. All patients received postoperative DV combined with a PD-1 inhibitor because they were either unsuitable for or declined platinum-based chemotherapy.

**TABLE 1:** Individual patient characteristics and postoperative outcomes of the UTUC cohort.

Patient	Age (years)	Primary site	Clinical/pathologic stage	HER2 IHC	Recorded treatment exposure	RFS status (mo)	Vital status/follow-up (mo)
Case-01	57	Ureter	cT2N0/pT2N0	3+	4 combination cycles +16 maintenance cycles	No recurrence (15.6)	Alive (15.6)
Case-02	64	Ureter	cT3N0/pT3N0	2+	4 combination cycles +1 maintenance cycle	No recurrence (17.9)	Alive (17.9)
Case-03	67	Renal pelvis	cT3N2/pT3N2	2+	4 combination cycles	Bladder recurrence (17.1)	Died (18.5)
Case-04	77	Ureter	cT2N0/pT2N0	3+	4 combination cycles	Bladder recurrence (6.4)	Alive (8.4)
Case-05	65	Ureter	cT3N0/pT4N0	2+	4 combination cycles	Bladder recurrence (3.1)	Died (33.4)
Case-06	70	Ureter	cT2N1/pT2N0	2+	4 combination cycles	No recurrence (24.8)	Alive (24.8)
Case-07	58	Ureter	cT2N1/pT2N1	2+	4 combination cycles	No recurrence (22.3)	Alive (22.3)

All patients had HER2 IHC scores of 2+ or 3+ (five cases of 2+ and two cases of 3+). PD-L1 expression was recorded as <1% for all seven patients. All patients completed four cycles of the DV plus PD-1 inhibitor regimen; two patients subsequently continued with PD-1 inhibitor monotherapy maintenance for 16 cycles and 1 cycle, respectively. For the remaining 5 patients, no further PD-1 maintenance therapy was recorded after the completion of 4 cycles of combination therapy.

### 3.2 Time-to-event outcomes

The median follow-up, estimated using the reverse Kaplan–Meier method, was 22.3 months. During follow-up, three cases of recurrence (42.9%; exact 95% CI: 9.9%–81.6%) occurred, all of which were local bladder recurrences; no cases of distant recurrence were confirmed. There were two deaths (28.6%; exact 95% CI: 3.7%–71.0%). Both patients had experienced local recurrence prior to death; based on available clinical records and clinical judgment, the causes of death were attributed to cardiovascular or cerebrovascular events. These deaths showed no clear association with the progression of UTUC or related anti-tumor therapies, and neither was classified as a treatment-related death. Kaplan-Meier estimates showed a 12-month postoperative RFS rate of 71.4% (95% CI: 25.8%–92.0%; 5 patients at risk), with the median RFS not reached. Due to the low number of deaths, OS data remain immature. Individual postoperative clinical courses, recurrence, and survival status are shown in Figure 2.

**FIGURE 2:**
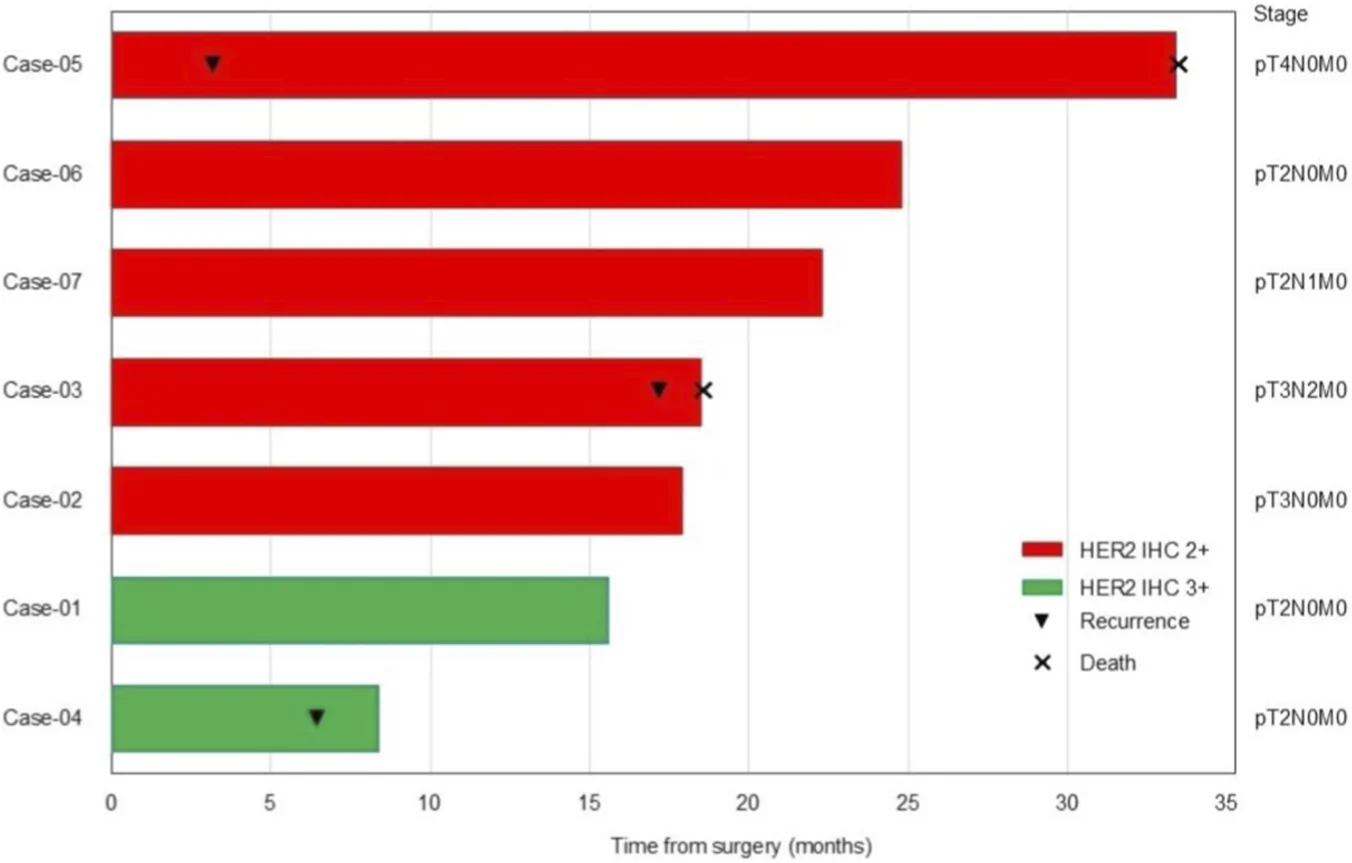
Swimmer plot showing the individual postoperative clinical courses of patients in the UTUC cohort. Red bars indicate HER2 IHC 2+, and green bars indicate HER2 IHC 3+; inverted triangles denote recurrence, and “ × ” marks denote death. The length of the bars represents the duration of postoperative follow-up from surgery to the last follow-up or death.

### 3.3 Safety

TRAEs of any grade occurred in four patients (57.1%); Grade ≥3 TRAEs occurred in two patients (28.6%) (Table 2). The most common event was fatigue (three patients). The two Grade ≥3 TRAEs were clinically diagnosed adrenal insufficiency and a demyelinating disorder; both events resolved after the suspension of postoperative treatment and appropriate clinical management. Additionally, one case of rash and one case of Grade 1 renal dysfunction were recorded. No Grade 4–5 TRAEs or treatment-related deaths were observed.

**TABLE 2:** Treatment-related adverse events (TRAEs) in the UTUC cohort.

TRAE	Any grade, n (%)	Grade 1–2, n	Grade ≥3, n
Fatigue	3 (42.9)	3	0
Rash	1 (14.3)	1	0
Renal dysfunction	1 (14.3)	1	0
Immune-related adrenal insufficiency	1 (14.3)	0	1
Demyelinating disorder	1 (14.3)	0	1
Patients with ≥1 TRAE	4 (57.1)	—	—
Patients with grade ≥3 TRAE	2 (28.6)	—	—

## 4 Discussion

This study describes seven patients with UTUC who underwent R0 resection and received postoperative combination therapy with DV and a PD-1 inhibitor, having either declined platinum-based chemotherapy or been deemed unsuitable for it. While all patients completed four cycles of the combination regimen, there was significant variation in subsequent PD-1 inhibitor maintenance therapy. During a median follow-up of 22.3 months, three recurrences and two deaths were observed; the median RFS was not reached. Given the very small sample size and the absence of a concurrent control group, these results cannot determine whether the regimen alters the natural history of UTUC; rather, the study’s primary value lies in characterizing actual treatment exposure, recurrence patterns, and safety profiles within this specific clinical population.

The three cases of initial recurrence observed in this study were all localized to the bladder; no distant recurrence has been confirmed to date. While this pattern of recurrence warrants attention, the findings must be interpreted with caution. Intravesical recurrence following surgery for UTUC is a significant clinical event in its own right; thus, the observed recurrence pattern likely reflects the characteristics of this small cohort rather than indicating that the combination of DV and a PD-1 inhibitor reduces the risk of distant metastasis. Given the small sample size (seven patients) and the relatively short median follow-up (22.3 months), the absence of observed distant metastasis is likely attributable to these limitations. Two patient deaths occurred during the study, both preceded by local recurrence; based on clinical records and assessment, these deaths were attributed to cardiovascular or cerebrovascular events rather than UTUC progression or complications from the anti-tumor therapy, and neither was classified as treatment-related. Consequently, these deaths should not be interpreted as tumor-related. With only two deaths recorded, the OS data remain highly immature. Future studies should distinguish between intravesical recurrence, other local or regional recurrences, and distant metastases, while evaluating disease-specific and overall survival outcomes over a longer follow-up period.

The heterogeneity in treatment exposure also merits attention. Although all seven patients completed four cycles of the DV plus PD-1 inhibitor regimen, the specific PD-1 inhibitors administered varied, including toripalimab and tislelizumab. Drug selection was not randomized or dictated by a predefined protocol but was instead based on real-world clinical practice at the participating centers. As the primary database did not systematically document the rationale for selecting a specific PD-1 inhibitor, it is impossible to determine whether clinical status, drug availability, or other factors influenced the choice; furthermore, this study could not compare the efficacy or safety profiles of toripalimab versus tislelizumab.

Significant heterogeneity was also observed regarding maintenance strategies following combination therapy. Only two patients received PD-1 inhibitor monotherapy for maintenance—receiving 16 cycles and one cycle, respectively—while no further maintenance therapy was recorded for the remaining five patients. Notably, neither of the two patients who received maintenance therapy experienced recurrence by the time of the last follow-up, whereas all three recurrences occurred in patients who did not receive maintenance therapy. However, this patient-level observation cannot be interpreted as evidence of a benefit from maintenance therapy, given that the treatment was not randomly assigned, the sample size was very small, and there were variations in treatment duration and follow-up periods. Furthermore, a causal relationship between maintenance therapy and recurrence or survival could not be assessed. Future prospective studies should pre-specify the cycles of combination therapy, the choice of PD-1 inhibitor, indications for maintenance therapy, and criteria for treatment discontinuation.

Existing perioperative studies on UTUC have confirmed the significant role of platinum-based chemotherapy and are exploring novel strategies involving immunotherapy and ADCs (Liu et al., 2025; Jayalath et al., 2026; Huang et al., 2025). The combination of DV and immunotherapy is currently being investigated for kidney-sparing treatment and metastatic disease in UTUC (Ye et al., 2025; Ng et al., 2025), and recent real-world studies have evaluated its use in the postoperative setting for high-risk UTUC (Wang et al., 2026). Therefore, this study should not be viewed as the first to demonstrate the use of DV combined with a PD-1 inhibitor for postoperative treatment; rather, its incremental value lies in providing detailed patient-level data—including treatment cycles, duration of maintenance therapy, patterns of recurrence, individual survival outcomes, and toxicity profiles—for a specific patient subgroup: those who underwent R0 resection, had no evidence of distant metastasis, and were either ineligible for or declined platinum-based chemotherapy.

All patients in the study were recorded as having HER2 IHC scores of 2+ or 3+; this result likely reflects an enrichment driven by clinical treatment selection based on HER2 expression rather than the true prevalence of HER2 expression in the overall UTUC population. It should be noted that the 0–3+ HER2 IHC classification framework used in this study references the ASCO/CAP scoring system for breast cancer (Wolff et al., 2013) and aligns with the 0–3+ classification framework adopted in the 2021 Chinese expert consensus on HER2 testing in urothelial carcinoma (Tumor Pathology Committee of Chinese Anti-Cancer Association, 2021); however, the ASCO/CAP criteria served primarily as a reference for semi-quantitative scoring in this study rather than as predictive criteria validated specifically for UTUC. HER2 expression can be influenced by factors such as testing methods, scoring systems, sampling sites, and tumor heterogeneity (Soria et al., 2017; Scherrer et al., 2022). Furthermore, as central pathology review was not conducted and systematic FISH confirmation was not performed for the five patients with IHC 2+ status, the HER2 results should be viewed primarily as background information regarding real-world treatment selection rather than as validated predictive biomarkers. Similarly, all patients exhibited PD-L1 expression levels <1%; the lack of inter-group variability precluded any assessment of the prognostic or predictive value of PD-L1 in this study.

Regarding safety, two patients experienced Grade ≥3 TRAEs: adrenal insufficiency and a demyelinating disorder. The adrenal insufficiency was clinically classified as an immune-related adverse event; the demyelinating disorder occurred during PD-1 inhibitor therapy and was potentially immune-related, though a causal relationship with the specific therapeutic agent could not be definitively established due to the lack of independent safety event adjudication. Both patients recovered following treatment interruption and appropriate management. Previous real-world studies of DV combined with PD-1 inhibitors generally indicate a manageable safety profile (Ng et al., 2025; Wang et al., 2026); however, the clinical significance of severe treatment-related toxicities may be more pronounced in the postoperative setting—specifically among patients who have undergone R0 resection and present with no clear distant metastases prior to treatment. Although no Grade 4–5 TRAEs or treatment-related deaths were observed in this study, future research should systematically monitor endocrine and neurological immune-related adverse events and document treatment interruptions, permanent discontinuations, and toxicity resolution to more comprehensively evaluate the benefit-risk balance of postoperative therapy.

This study has several unavoidable limitations. First, the sample size and number of events were very small; the 95% CI for 12-month RFS was wide, and with only two deaths recorded, OS data remain immature. Furthermore, the median follow-up duration in this study was only 22.3 months, which is relatively limited for evaluating long-term recurrence patterns; thus, the possibility of late local or distant recurrence occurring beyond the current observation period cannot be ruled out. Consequently, the reported 12-month RFS and the absence of observed distant recurrence should be interpreted as relatively early postoperative findings rather than evidence of long-term, durable disease control. Longer follow-up is required to more fully evaluate late recurrence and long-term survival outcomes. Second, the absence of a concurrent control group introduced confounding by indication, selection bias, and inter-center variability, precluding causal inference. Third, although all patients underwent R0 resection and lacked variant histology, heterogeneity persisted regarding tumor location and pathological stage, and specific surgical approaches were not recorded as standardized, structured variables. Fourth, the PD-1 inhibitors administered to patients were not uniform, comprising agents such as toripalimab and tislelizumab; and the choice of drug was neither randomized nor determined by a predefined study protocol. Given that the retrospective database did not standardize the recording of the full rationale for selecting specific PD-1 inhibitors for each patient, combined with the very small sample size, this study could not assess whether different PD-1 inhibitors differentially affected efficacy or safety. Consequently, the observed results should be interpreted as an exploratory overview of the treatment strategy combining DV with PD-1 inhibitors, rather than a comparison of the efficacy of different PD-1 agents. Furthermore, the strategy for maintenance therapy following the completion of four cycles of combination therapy was not standardized: only two patients subsequently received PD-1 inhibitor monotherapy for maintenance—one for 16 cycles and the other for a single cycle. As the specific reasons for initiating, omitting, or discontinuing maintenance therapy were not systematically recorded, it is impossible to determine whether exposure to maintenance therapy influenced recurrence or safety outcomes. This heterogeneity in treatment duration further limits the interpretation of the current findings. Furthermore, data regarding cycle-by-cycle dose adjustments, treatment delays, and reasons for discontinuation were incomplete. Fifth, the study lacked central review of imaging and pathology. HER2 assessment was based on routine IHC testing at individual centers; central pathology review was not performed, nor was FISH routinely used to confirm HER2 status. This limitation is particularly significant given that five of the seven patients had an IHC score of 2+; in the absence of further confirmatory testing, the possibility of HER2 status misclassification cannot be ruled out, which may affect the interpretation of patient selection and treatment outcomes. Although the SP263 assay system was used for PD-L1 assessment in all cases, the retrospective databases did not fully preserve detailed scoring components or specific instrument parameters. Consequently, while all patients were recorded as having PD-L1 expression <1%, we could not fully verify the consistency of testing methodology across different patients and centers, nor could we further interpret PD-L1 status in terms of prognosis or predictive value regarding treatment efficacy. Finally, the assessment of adverse events relied on retrospective medical records and lacked an independent adjudication committee; thus, the incidence of mild adverse events may have been underestimated.

Given these limitations, this case series should primarily be viewed as a descriptive, hypothesis-generating study rather than evidence supporting the efficacy of the postoperative DV plus PD-1 inhibitor regimen. The study’s main contribution lies in documenting the real-world feasibility, heterogeneity of treatment exposure, postoperative clinical events, and clinically significant toxicities associated with this treatment strategy in a small cohort of patients who did not receive platinum-based chemotherapy. Future research should involve multicenter studies featuring prospective registration, concurrent standard-of-care control groups, standardized HER2 testing, precise documentation of treatment initiation dates and per-cycle exposure, and systematic monitoring of immune-related toxicities, and sufficiently long-term follow-up to evaluate late recurrence and long-term survival outcomes; statistical approaches such as propensity score analysis, weighted survival models, or multivariable adjustment should be considered only after a sufficient number of events has been accumulated.

## 5 Conclusion

In this retrospective, exploratory case series, all patients underwent complete R0 resection and were diagnosed with pure urothelial carcinoma. This study describes treatment exposure, patterns of recurrence, individual clinical courses, and safety outcomes associated with the use of adjuvant DV combined with PD-1 inhibitors in selected patients with UTUC, while also identifying immune-related adverse events that warrant close attention. However, given the lack of a control group and the very small sample size, these findings require further validation.

## Data Availability

The original contributions presented in the study are included in the article, further inquiries can be directed to the corresponding authors.

## References

[B1] Bellmunt J. Hussain M. Gschwend J. E. Albers P. Oudard S. Castellano D. (2021). IMvigor010 study group. Adjuvant atezolizumab versus observation in muscle-invasive urothelial carcinoma (IMvigor010): a multicentre, open-label, randomised, phase 3 trial. Lancet Oncol. 22 (4), 525–537. 10.1016/S1470-2045(21)00004-8 33721560 PMC8495594

[B2] Birtle A. Johnson M. Chester J. Jones R. Dolling D. Bryan R. T. (2020). Adjuvant chemotherapy in upper tract urothelial carcinoma (the POUT trial): a phase 3, open-label, randomised controlled trial. Lancet. 395 (10232), 1268–1277. 10.1016/S0140-6736(20)30415-3 32145825 PMC7181180

[B3] Chen A. P. Setser A. Anadkat M. J. Cotliar J. Olsen E. A. Garden B. C. (2012). Grading dermatologic adverse events of cancer treatments: the common terminology criteria for adverse events version 4.0. J. Am. Acad. Dermatol 67 (5), 1025–1039. 10.1016/j.jaad.2012.02.010 22502948

[B4] Huang J. Cai X. Ng C. Luo Y. Chen Q. Wang Z. (2025). A phase 2 study of tislelizumab as neoadjuvant treatment of cisplatin-ineligible high-risk upper tract urothelial carcinoma. J. Urol. 213 (6), 739–752. 10.1097/JU.0000000000004475 39999445 PMC12708035

[B5] Iwata T. N. Ishii C. Ishida S. Ogitani Y. Wada T. Agatsuma T. (2018). A HER2-targeting antibody–drug conjugate, trastuzumab deruxtecan (DS-8201a), enhances antitumor immunity in a mouse model. Mol. Cancer Ther. 17 (7), 1494–1503. 10.1158/1535-7163.MCT-17-0749 29703841

[B6] Jayalath V. H. Assel M. Iyer G. Niglio S. Bochner B. H. Dalbagni G. (2026). Neoadjuvant chemotherapy for high-risk localized upper tract urothelial carcinoma: final long-term outcomes from a phase 2 clinical trial and an expanded cohort. Eur. Urol. 89 (2), 108–113. 10.1016/j.eururo.2025.08.012 41006098

[B7] Liu Y. Song Y. Li J. Rui C. Qin C. Xu T. (2025). Comparative efficacy of adjuvant chemotherapy and immunotherapy after radical surgery for upper tract urothelial carcinoma: a systematic review and meta-analysis. Urol. Oncol. 43 (9), 473–484. 10.1016/j.urolonc.2025.03.023 40414745

[B8] Masson-Lecomte A. Birtle A. Pradere B. Capoun O. Compérat E. Domínguez-Escrig J. L. (2025). European association of urology guidelines on upper urinary tract urothelial carcinoma: summary of the 2025 update. Eur. Urol. 87 (6), 697–716. 10.1016/j.eururo.2025.02.023 40118741

[B9] Ng C. Jing T. Yu S. Ye J. Zhang S. Jia Z. (2025). The efficacy and safety of disitamab vedotin combined with immune checkpoint inhibitors in metastatic upper tract urothelial carcinoma: a multicenter real-world study. Cancer Immunol. Immunother. 74 (10), 304. 10.1007/s00262-025-04154-5 40944727 PMC12433416

[B10] Scherrer E. Kang A. Bloudek L. M. Koshkin V. S. (2022). HER2 expression in urothelial carcinoma, a systematic literature review. Front. Oncol. 12, 1011885. 10.3389/fonc.2022.1011885 36338710 PMC9635895

[B11] Schemper M. Smith T. L. (1996). A note on quantifying follow-up in studies of failure time. Control Clin. Trials 17 (4), 343–346. 10.1016/0197-2456(96)00075-x 8889347

[B12] Sheng X. Wang L. He Z. Shi Y. Luo H. Han W. (2024). Efficacy and safety of disitamab vedotin in patients with human epidermal growth factor receptor 2-positive locally advanced or metastatic urothelial carcinoma: a combined analysis of two phase II clinical trials. J. Clin. Oncol. 42 (12), 1391–1402. 10.1200/jco.22.02912 37988648 PMC11095880

[B13] Sheng X. Zeng G. Zhang C. Zhang Q. Bian J. Niu H. (2025). RC48-C016 trial investigators. Disitamab vedotin plus toripalimab in HER2-expressing advanced urothelial cancer. N. Engl. J. Med. 393 (23), 2324–2337. 10.1056/nejmoa2511648 41124210

[B14] Soria F. Moschini M. Haitel A. Wirth G. J. Karam J. A. Wood C. G. (2017). HER2 overexpression is associated with worse outcomes in patients with upper tract urothelial carcinoma (UTUC). World J. Urol. 35 (2), 251–259. 10.1007/s00345-016-1871-x 27272502

[B15] Tumor Pathology Committee of Chinese Anti-Cancer Association; Expert Committee on Urothelial Carcinoma of Chinese Society of Clinical Oncology (2021). Clinical pathological expert consensus on HER-2 testing in urothelial carcinoma in China. Zhonghua Zhong Liu Za Zhi 43 (10), 1001–1006. 10.3760/cma.j.cn112152-20210809-00597 34695889

[B16] Wang C. He S. Li D. Zhang B. Dai Y. Li C. (2026). Adjuvant disitamab vedotin plus PD-1 blockade and gemcitabine/cisplatin in high-risk upper tract urothelial carcinoma: a two-stage real-world comparative study. Front. Immunol. 17, 1809094. 10.3389/fimmu.2026.1809094 42382757 PMC13315034

[B17] Wolff A. C. Hammond M. E. H. Hicks D. G. Dowsett M. McShane L. M. Allison K. H. (2013). Recommendations for human epidermal growth factor receptor 2 testing in breast cancer: American society of clinical oncology/college of American pathologists clinical practice guideline update. J. Clin. Oncol. 31 (31), 3997–4013. 10.1200/jco.2013.50.9984 24101045

[B18] Ye J. Chen Z. Liao X. Zhang S. Tu X. Wang Q. (2025). Endoscopic thulium laser ablation with disitamab vedotin and immunotherapy in high-risk upper tract urothelial carcinoma: a prospective pilot study of a novel kidney-sparing strategy. Eur. Urol. Oncol. 8 (6), 1607–1616. 10.1016/j.euo.2025.09.015 41107105

[B19] Zhou L. Yang K. W. Zhang S. Yan X. Q. Li S. M. Xu H. Y. (2025). Disitamab vedotin plus toripalimab in patients with locally advanced or metastatic urothelial carcinoma (RC48-C014): a phase Ib/II dose-escalation and dose-expansion study. Ann. Oncol. 36 (3), 331–339. 10.1016/j.annonc.2024.12.002 39662628

